# Impact of food, beverage, and alcohol brand marketing on consumptive behaviors and health in children and adults: A systematic review and meta‐analysis

**DOI:** 10.1111/obr.13932

**Published:** 2025-04-14

**Authors:** Emma Boyland, Nicholas Davies, Moon Wilton, Andrew Jones, Michelle Maden, Ffion Curtis, Rebecca Evans, Amy Finlay, Lauren McGale, Caroline Cerny, Nika Pajda, Abigail K. Rose

**Affiliations:** ^1^ Department of Psychology University of Liverpool Liverpool UK; ^2^ Liverpool John Moore's University Liverpool UK; ^3^ Liverpool Reviews and Implementation Group University of Liverpool Liverpool UK; ^4^ Edge Hill University Ormskirk UK; ^5^ Bite Back 2030 London UK

**Keywords:** alcohol, beverage, brand, food, marketing, meta‐analysis, systematic review

## Abstract

Exposure to unhealthy food, beverage, and alcohol marketing can contribute to inadequate diet and excess alcohol consumption, both risk factors for diet‐related non‐communicable diseases including obesity and cancer. By not featuring specific products, brand‐only marketing strategies circumvent restrictions that assess healthiness at the product level and restrict accordingly. Currently, there is no global or national government policy that explicitly addresses brand marketing for unhealthy products linked to diet‐related non‐communicable diseases. This systematic review and meta‐analysis synthesizes contemporary evidence on the effects of food, beverage, and alcohol brand‐marketing on diet‐related cognitive outcomes (preference, choice), diet‐related behavioral outcomes (purchase requests, purchase, consumption), and health‐related outcomes (body weight, body mass index, obesity) in children and adults. Included studies manipulated acute marketing exposure, with at least one brand‐only marketing condition. Fourteen databases were searched (including MEDLINE and PubMed) for articles published from January 2004 to February 2024. Nineteen eligible studies were identified and assessed for bias; five were included in the meta‐analysis assessing effects on consumption. Findings from the review suggest brand marketing for food, beverages, and alcohol can influence preference, choice, and purchase intent. The meta‐analysis found no evidence of a significant effect of brand‐only marketing on consumption. Overall, evidence was limited and of mixed quality so further robust research is needed to inform regulatory action. Government policies for reducing brand‐only marketing are needed to protect vulnerable populations from brand marketing promoting unhealthy consumption behaviors that increase the risk of non‐communicable disease.

## 1 INTRODUCTION

Poor diet and excess alcohol consumption are both major global public health concerns across the life course, with nutritional deficiencies, high body mass index, and alcohol use key risk factors for poor health and mortality.^1^ The marketing of unhealthy food and non‐alcoholic sugary beverages (hereafter: food) and alcohol significantly influences consumption behaviors in both adults and children.^2^, ^3^, ^4^, ^5^, ^6^ Marketing is defined by the World Health Organization (WHO) as ‘any form of commercial communication, message or action that acts to advertise or otherwise promote a product or service, or its related brand, and is designed to increase, or has the effect of increasing, the recognition, appeal and/or consumption of products or services’.^7^ Food marketing has been causally linked to childhood weight gain and obesity,^8^ while alcohol marketing is associated with initiation of drinking and risk of hazardous drinking in young people^9^, ^10^ and alcohol use in adults.^11^ Consequently, the WHO advocates for stringent restrictions on such marketing to reduce the global burden of non‐communicable diseases.^7^, ^12^, ^13^

Despite several countries imposing restrictions,^14^, ^15^ both adults and young people continue to be exposed to extensive unhealthy food and alcohol marketing,^16^ particularly via digital media.^17^, ^18^ This has been partly attributed to an over‐reliance on ineffective self‐regulatory measures^15^, ^19^ and greater use of brand marketing by corporations to build brand awareness and brand loyalty to generate brand equity and maximize sales, revenue, and profits for businesses globally. Brand equity (the ‘value‐added’ a product acquires through connection with a brand name) is a key determinant of consumers' brand attachments,^20^ preferences,^21^ and purchase behaviors.^22^ Brand marketing is defined as “the approach used by companies to promote and establish a brand in a market by creating a unique identity, values, and perceptions that differentiate it from competitors”.^23^ In practice, this may present as the promotion of branding elements (e.g., logos, marks, characters, colors, or straplines that are directly associated with a particular product, product range, or company) but with no identifiable product.^24^ Recent evidence suggests that around 40% of food advertising is now for brands rather than specific products and 89% of the top 20 companies' brand sales in the UK were classified as “unhealthy” according to WHO criteria.^25^ Additional potentially harmful brand‐related strategies include marketing zero‐alcohol products^26^, ^27^ (which often share brand iconography with ‘regular strength’ products^28^) and other ‘stealth marketing’ tactics, including ‘alibi’ marketing,^29^ whereby key components of a brand's identity (such as colors, fonts, and slogans) are presented without explicitly mentioning the core brand name (exemplified by Carlsberg's ‘Probably …’^30^ and McDonalds' ‘eyebrows’ campaigns).^31^

To date, food advertising restrictions have exclusively focused on product‐based advertising, often using nutrient profile models to determine what can and cannot be marketed.^15^ This requires an identifiable product to be featured in the advertisement. Food and alcohol marketers are increasingly using brand marketing to these circumvent product‐based restrictions^32^ and self‐regulatory industry initiatives, such as the EU Pledge and the International Food & Beverage Alliance's Global Policy on Marketing Communications to Children, do not tend to restrict brand marketing strategies such as brand mascots and as such do not meet their stated commitments to responsible marketing to children.^33^, ^34^ Therefore, these activities and their implications require further consideration. There is yet to be a policy, globally, that restricts brand‐only marketing of unhealthy commodities. In this way, there appear to be commonalities with the stages of tobacco regulation, whereby product‐based restrictions long preceded brand‐based approaches.^35^

It is not straightforward to define what constitutes a brand, which adds further complexity to the development of regulation. Brand architecture has a complicated structure, whereby a single corporate brand may own several product brands (which may each have multiple sub‐brands).^36^ The master brand for a business (i.e., the main and most recognizable brand for customers^36^) may have the same name as a product brand (e.g., Mars Inc., Mars Bar) or not (e.g., Coca‐Cola Company, Dr Pepper). But it is clear that brands have valuable representational and rhetorical power inherent in the societal, cultural, and ideological meaning and value they hold, and they can be considered from multiple different perspectives including that of the corporation (focusing on brand image and value) and the consumer (focusing on how brands relate to identities and self‐concepts).^37^ Investment in brand‐building marketing to leverage that power is critical for companies as they seek greater market share in the long term, including via customer acquisition from competitors in the shorter term.^38^ To do this, marketing develops and builds brand awareness (visibility, recognition, and embedding in consumer consciousness), brand preference, and brand loyalty, which precede purchase behavior and sales growth.^38^, ^39^ Central to this is the creation of positive brand affect^40^ and the emotional priming of consumers to increase their propensity to purchase products from that brand.^38^ Given the prevalence of unhealthy food and alcohol brand marketing globally,^17^, ^41^ outcomes such as increased purchase propensity have clear relevance to diet quality, alcohol consumption, and obesity risk, and are of concern for public health.

Brands use multiple marketing strategies to encourage consumers to recognize and positively associate with their brands, for example, promotional characters^42^ and endorsement and co‐branding by music^43^ and sport^44^ celebrities are often used to appeal to children and young people. Increasingly, this includes digital gaming media and marketing of products such as energy drinks and snacks by influencers with huge numbers of followers.^45^ Emotional attachments (parasocial relationships) can form between young people and promotional characters, celebrities, and influencers based on their credibility, familiarity, and perceived accessibility.^46^

Studies have shown that children as young as three can recognize brand logos and associate them with specific products, even for adult‐directed products (e.g., cigarettes and alcohol^47^, ^48^). Recognition of food brand imagery is also significantly associated with higher body mass index in children.^49^, ^50^ Children also demonstrate strong positive affect towards brands,^51^ and their beliefs about brand‐associated personalities indicate the potential normative social influence of brand‐driven marketing.^52^ Adolescents, in particular, are known to actively engage with brands and brand marketing online.^53^, ^54^ Such engagement has been demonstrated to affect their desire to consume unhealthy foods,^55^ and may be more strongly associated with purchase and consumption behaviors than exposure alone.^54^, ^56^ Despite most research focusing on the impact of advertising on young people,^57^, ^58^ mechanistic studies indicate that emotional attachments to brands can form at a neurological level in adults,^59^ with exposure to brand imagery activating brain regions relating to emotional, as well as visual, processing.^60^

Given the increasing use of food and alcohol brand marketing, it is important to understand the specific impact of this (as distinct from product‐based advertising) on consumptive behaviors and health. This insight should inform evidence‐based policies that advocate for broader and stricter regulation of food and alcohol marketing, aimed at reducing related health risks and harm. Therefore, the aim of this review was to identify and synthesize evidence of the impact of brand‐only marketing of food and alcohol on diet‐related cognitive, behavioral, and health outcomes in children and adults.

## 2 METHODS

The research question to be addressed was: what is the effect in children and adults on the outcomes of interest of exposure to brand‐only marketing (i.e., with no product featured) for foods and alcohol compared with exposure to a relevant comparator? This systematic review and meta‐analysis was pre‐registered with PROSPERO (registration number: CRD40244506357, available from https://www.crd.york.ac.uk/prospero/display_record.php?RecordID=506357) and is reported in accordance with the PRISMA guidelines.^61^

### 2.1 Search strategy

A comprehensive search strategy was developed and executed with the support of experienced information specialists (MM, FC; see Table 1). Searches were conducted in MEDLINE, PubMed, Embase, Cochrane CENTRAL, CINAHL, Web of Science (all platforms), ERIC, Business Source Complete, Emerald, HMIC, Social Policy, and Practice, Google Scholar (targeted search – i.e., an advanced search using key terms searched within the title field only), Institutional Repository of Information Sharing (IRIS), and Communication & Mass Media Complete for articles published from 1 January 2004 (to maximize the relevance of the evidence to the contemporary commercial and media environment) until 24 February 2024. Both subject headings and free‐text terms were combined. These searches were supplemented by hand‐searching reference lists of retrieved systematic reviews and contact with topic experts. We used EndNote X9^62^ and Rayyan^63^ for citation management.

**TABLE 1:** Search strategy.

**Population terms**	Human NOT animals
**AND**	
**Behavior terms**	Food* OR diet* OR snack* OR nutrition* OR fast food* OR beverage* OR drink* OR tea OR milk OR juice OR alcohol* OR non‐alcohol* OR low‐alcohol*
**AND**	
**Marketing terms**	Advert OR advergam* OR sponsor* OR promot* OR market* OR commercial
**AND**	
**Branding terms**	Brand* OR unbrand* OR logo* OR slogan* OR food‐brand* OR beverage‐brand* OR alcohol‐brand* or alcoholic*‐brand

### 2.2 Eligibility criteria

See Table 2. The criteria for inclusion were: (i) studies of healthy (systemic disease‐free) child (0–18y) and/or adult (18y+) populations; (ii) manipulated acute marketing exposure, including at least one condition in which participants were exposed to brand‐only (i.e., not featuring identifiable products) commercial marketing (as defined by the WHO^7^) for foods or alcohol and another condition with a relevant control stimulus (full details are given in Supplement Table S8 but example appropriate comparators for a food brand exposure would be a food product with no brand imagery, brand imagery for a generic/supermarket brand of the same product type, brand imagery for a brand unrelated to consumption such as toys, or no marketing); (iii) reported measured or self‐reported data on one or more of the outcomes of interest (Supplement Table S9) derived from the Hierarchy of Unhealthy Food Promotion Effects model which provides a conceptual framework for evidence‐based understanding of the relationship between exposure to marketing and poor health.^21^ Specifically, these were diet‐related cognitive outcomes (brand and/or product preference, brand and/or product choice/intended choice), diet‐related behavioral outcomes (brand and/or product requests/intended requests, brand and/or product purchasing/intended purchase, brand and/or product consumption/intended consumption), and health‐related outcomes (body weight/body mass index, obesity); (iv) published in English in a peer‐reviewed journal.

**TABLE 2:** PICO table.

Criteria	Determinants
*Populations*	Children (0–18 years) and adults (19 + years). If possible, consider differences by equity characteristics (socioeconomic status, country of residence, age, gender, etc.).
*Interventions*	Exposure to brand‐only marketing (i.e., not featuring products) for food, beverages, or alcoholic beverages.
*Comparison*	Exposure to appropriate comparator (see Table S8).
*Outcomes* *(See Table* *S9* *for definitions)*	Food, beverage, and alcoholic beverage consumption or intended consumption. Food, beverage, and alcoholic beverage choice or intended choice. Food, beverage, and alcoholic beverage purchasing/sales (by adults, by children, or on behalf of children) or intended purchasing. Food, beverage, and alcoholic beverage preferences. Product requests by adults or children (e.g. “pester power”) or intended requests. Body weight/body mass index/obesity.

### 2.3 Study selection and data extraction

Two reviewers from a pool of three (EB, ND, MW) independently screened studies against the inclusion criteria; assessing titles and abstracts to identify potentially relevant studies. Two reviewers (ND and another reviewer from a pool of five: EB, RE, AF, LM, AR) independently reviewed full texts. Data extracted for eligible records included lead author, publication date, country, study design, population characteristics, commodity category (food, alcohol) and related information (e.g., brand names or healthiness judgments undertaken by authors), marketing type (e.g., TV advertisement, digital marketing, packaging, sponsorship), outcome statistics (descriptive and inferential), funding source and reported conflicts of interest. One reviewer (ND) extracted the relevant data using a pre‐defined and piloted template in Excel (MW), these data were cross‐checked by a second reviewer (EB). For both study selection and data extraction, disagreements were resolved through consensus.

### 2.4 Quality assessment

Included studies were assessed and critically appraised using appropriate tools for the study designs (Risk of Bias 2^64^ for RCTs and Newcastle‐Ottawa scale^65^ for experimental designs, as in^2^). The quality of the included studies was assessed by one reviewer (ND), and independently checked for agreement by a second (EB). Any disagreements would have been resolved through consensus, but no such disagreements arose.

### 2.5 Data synthesis and analysis

Studies were organized by the commodity category of brands (food, alcohol) to aid between‐study comparisons. Data extracted was summarized in tables and described in a narrative synthesis in accordance with guidelines.^66^

Studies with comparable outcomes were pooled using multi‐level, random effects meta‐analyses with Restricted Maximum Likelihood Estimators to estimate an overall effect size.^67^ Meta‐analysis was conducted for consumption because studies with comparable outcomes provided at least four effect sizes.^68^ Study 3 from Werle et al, (2016)^69^ contributed two effect sizes to the analysis, the plain packaging vs. each of the original and the lighter packaging group.

These studies provided continuous data, so the standardized mean difference (SMD) was computed and compared. Heterogeneity was investigated using the I^2^ statistic, where higher percentages indicate greater heterogeneity. Publication bias was explored through visual inspection of funnel plots as well as trim‐and‐fill analyses, as recommended by Cochrane guidance. Analyses were conducted in R (Metafor).^70^

### 2.6 Data and code availability

The data used in the meta‐analysis were obtained from the selected studies (Tables S1 and S2). For a few studies, additional data/information was supplied by the authors on request (Supplement, Table S10). Analyzed data and the R code required to reproduce the meta‐analysis are publicly available via the Open Science Framework (https://osf.io/8ma7z/?view_only=fd97a8292c4841ba9fd481876dc6077f).

## 3 RESULTS

### 3.1 Description of included studies

**FIGURE 1:**
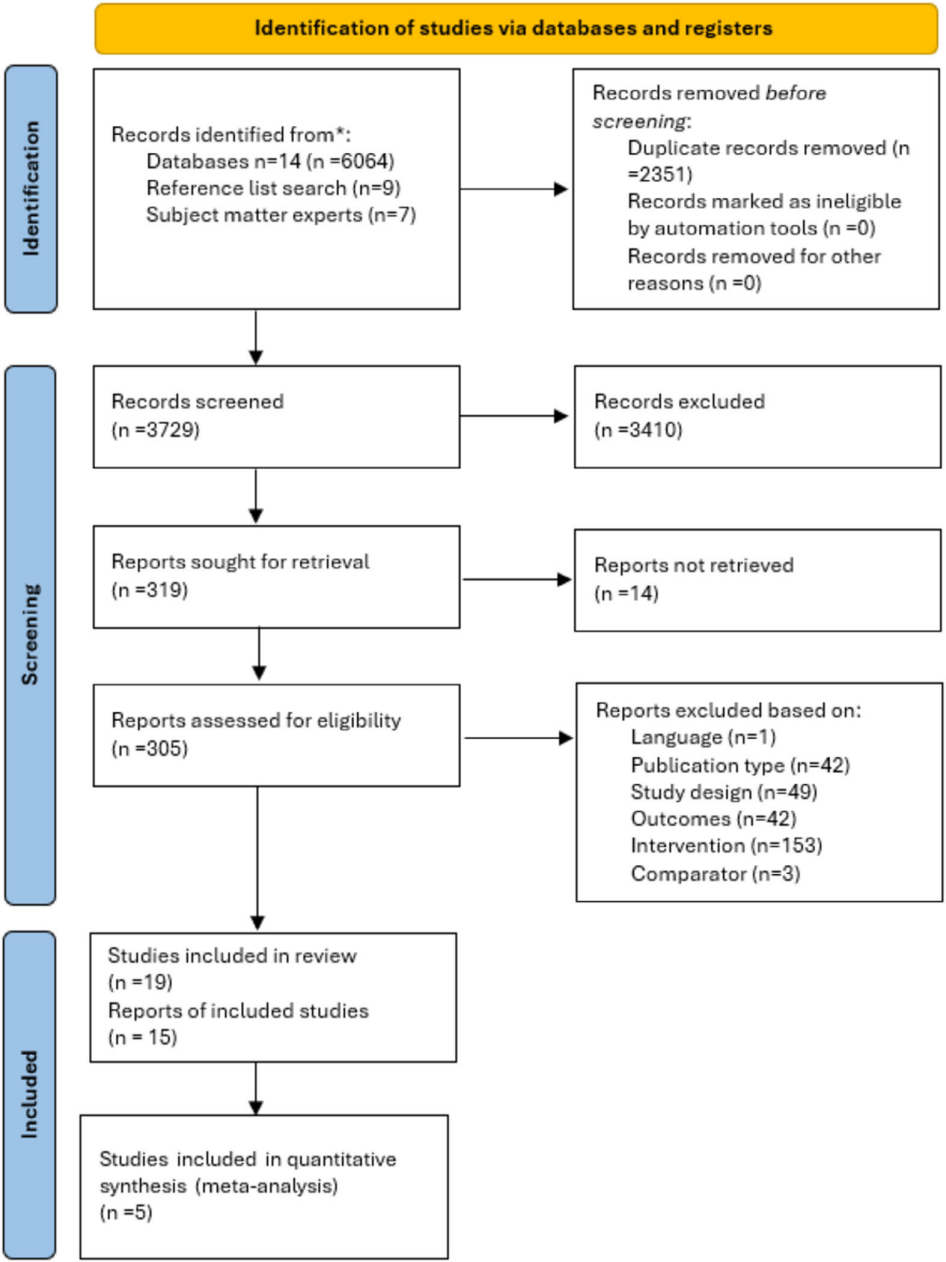
Preferred reporting items for systematic reviews and meta‐analyses (PRISMA) flow diagram. See Methods and supplementary information for the details of the systematic literature review approach, protocol and selected studies.

Searches identified 3729 (de‐duplicated) records and a total of 19 studies (from 15 articles) were eligible for inclusion in the review (Figure 1). The same data was analyzed in study 1 of Keller et al, (2012)^71^ and in Forman et al, (2009)^72^ so this was considered a single linked study. Sixteen studies examined food brands and three examined alcohol brands.

Of the eligible studies, 16 were randomized controlled trials (RCTs) and 3 were non‐randomized studies (NRS). Five studies were conducted in the USA, three in Australia, three in the UK, two in Chile, and one each in Brazil, Canada, and Poland. Twelve included adult (18 years and over) participants and seven included children (<18 years). Sample sizes ranged from 40 to 1132 participants, and mean age ranged from 3.8 years to 27.8 years. Brand‐only marketing stimuli varied between studies; 11 studies manipulated branding through packaging and four studies used sponsorship‐related stimuli (e.g., sports merchandise). The remaining studies used signage or digital game stimuli. The most common outcomes were ‘preference’ (nine studies assessed preference) and ‘intake’ (seven studies), followed by ‘choice’ (five studies) and ‘purchase’ (two studies). For full study characteristics, see Supporting Information (Tables S1–S3).

### 3.2 Narrative synthesis

#### 3.2.1 Studies assessing food brand marketing in children

Seven studies examined the effect of food brand marketing exposure with children and all studies used an RCT design (see Table S1, Supporting Information). Six of these studies manipulated brand marketing through packaging, comparing exposure to branded packaging (e.g. featuring logos or brand equity characters) to plain unbranded packaged items. The studies assessing preference and choice‐related outcomes generally showed that children prefer (or are more likely to choose) branded food items compared to unbranded items.^73^, ^74^, ^75^ Two studies examining the effect of branded packaging on consumption found more mixed results. One study found that branded packaging exposure led to increased food intake, subgroup analyses showed that this was driven by an effect found in females but not males.^71^ A similar study found no effect between the branded and unbranded conditions overall.^71^, ^72^ This study, however, did demonstrate subgroup differences where children with overweight appeared more likely to consume more calories in the branded compared to the unbranded condition. The quality of evidence was mixed, with all studies assessed as having some concerns about the risk of bias assessment (see Table S4 and Figure S1).

The remaining study manipulated brand marketing through sports sponsorship, with sports merchandise branded either with unhealthy or healthier food brand logos.^76^ The control groups were either exposed to non‐food‐related sponsors (e.g., a travel company) or branding with an obesity prevention campaign. Exposure to unhealthy food sponsors did not influence brand preference compared to the non‐food brand control group. Similarly, exposure to healthier sponsors did not influence brand preference for healthier brands compared to the non‐food brand control group. Children exposed to the healthier branding, however, showed reduced preference for unhealthy sponsor brands. The study had some concerns of bias (see Figure S1).

#### 3.2.2 Studies assessing food brand marketing in adults

Nine studies examined brand marketing of food brands, and similarly to the research with children, the majority (six studies) manipulated brand‐only exposure through packaging^69^, ^77^, ^78^, ^79^ (See Table S2, Supporting Information). Most studies (n = 6) used randomized study designs.

Adults generally reported a greater preference for beverages when they were exposed to branding information rather than when evaluating them blind.^77^, ^79^ Similarly, consumers had a higher intention to purchase beverage brands after being exposed to branding information, particularly for well‐known national brands vs regional or store brands.^78^ The evidence quality was low as indicated by scores of three or less in the quality assessment.

In accordance with the childhood research, exposure to branded vs unbranded packaging appeared to have mixed effects on consumption. Three linked studies by Werle et al (2016) found that while branded packaging increased intended consumption, relative to plain packaging, these effects were not present when the studies measured actual food intake in calories.^69^ The second study by Werle et al^69^ found no significant differences in overall consumption when food (chocolate) was served in its original branded packaging vs plain unbranded packaging. However, males in the plain packaging condition showed significantly greater consumption than in the branded packaging condition. The third and final study by Werle et al compared exposure to the chocolate confection in their original branded packaging to the chocolate confection branded as lighter and lower in fat, as well as the control condition where the chocolates were served in plain unbranded packaging. Here, participants consumed more in the unbranded and reduced fat conditions compared to the original branded condition. Gender differences were also found, with males consuming more in the unbranded and low‐fat conditions, and females consuming more in the low fat compared to the original, branded condition. The quality of evidence was mixed, as the studies were evaluated as having some concerns about the risk of bias assessment.

Dixon et al, (2018)^80^ examined brand marketing through sports sponsorship, where sponsors were unhealthy or healthy food brands compared with either non‐food and drink‐related brands or sponsorship by an obesity prevention public health campaign. Exposure to the unhealthy food sponsors did not influence preferences, however, exposure to healthier sponsors increased preference for healthier food sponsor brands compared to the non‐food sponsorship condition. The quality of the evidence was mixed, with this study being judged as having some concerns in the risk of bias assessment.

Two studies reported in Farrar et al, (2022)^81^ manipulated brand marketing exposure through priming tasks involving logos. In the first study, the priming task involved branding for two different food brands, compared to no food‐related branding. Participants in the food brand priming conditions were no more likely than participants exposed to no logos to select a product from one of the primed brands. In the second study, the priming task included a greater range of unhealthy food‐related logos compared to non‐food‐related logos. There were no significant differences in choosing unhealthy food brands between the experimental and control conditions. Both studies were evaluated as having some concerns about the risk of bias assessment, indicating a degree of uncertainty in the quality of the evidence.

#### 3.2.3 Studies assessing alcohol brand marketing in adults

Three studies examined brand marketing for alcohol with adult participants (see Table S3, Supporting Information). Two studies by the same primary author used sports sponsorship stimuli where sporting events were either sponsored by beer brand logos or by non‐alcohol (sports brand) logos.^82^, ^83^ The remaining study featured a digital game that either featured beer branding or non‐alcohol‐related branding (energy drinks).^84^ Only one study found a significant effect of brand‐only exposure, where alcohol sponsorship increased intention to purchase.^82^ The remaining studies examined intention to consume alcohol^83^ or alcohol choice^84^ and found no significant effects between participants exposed to alcohol branding and those exposed to unrelated brands. The quality of evidence was low, with two studies assessed as having a high risk of bias and one having some concerns of bias.

### 3.3 Meta‐analysis

Five studies assessed the effect of brand‐only marketing on diet‐related behavioral outcomes (specifically, actual intake and intended intake) and provided sufficient data for inclusion in meta‐analysis.^69^, ^71^, ^72^ All five studies examined branded versus unbranded packaging for food brand items. There was no evidence of a significant effect of brand‐only marketing on consumption (SMD = 0.30 [95%CI = ‐0.07, 0.67], p = 0.11, I^2=^80.75%). Figure S2 (Supplementary Information) shows the individual SMDs for each study included in the model. There was some evidence of publication bias, as indicated by Trim and Fill imputation (see Table S6) though this should be interpreted with caution due to the small number of studies.

There was not sufficient data to compute meta‐analyses for the other outcomes assessed across the trials.

## 4 DISCUSSION

The current study synthesized evidence from 19 studies of the impact of acute experimental exposure to brand‐only marketing for unhealthy food or alcohol on a variety of behavioral and health outcomes in children and adults. Results suggest that brand marketing can influence diet‐related cognitive and behavioral outcomes (specifically preference, choice, purchase intent) for foods and/or alcohol, but these effects have not been consistently found. Similar to the evidence on product advertising,^85^, ^86^ some sub‐group differences were evident, specifically greater responding to food brand marketing in children with overweight (relative to healthy weight peers) and girls (relative to boys). Studies investigating the effects of brand‐only marketing on actual consumption of food or alcohol were limited, with a meta‐analysis of five studies showing no overall effect. No studies examined health‐related outcomes.

Notably, most studies in the current review experimentally manipulated food marketing exposure using packaging stimuli (e.g., comparing packaging with and without brand imagery). This does not adequately reflect modern marketing exposure (nor, therefore, its likely impact), particularly with respect to digital media. Nevertheless, commercial strategies and features on food packaging, including promotional characters and logos, are thought to be a powerful form of marketing^87^ that attracts attention, shapes associations, and influence purchase decisions.^88^ Use of on‐pack marketing strategies has previously been shown to be more prevalent for less healthy products^89^ and can impact diet‐related behaviors in children and adolescents.^90^, ^91^ In 2016, Chile implemented a law mandating front‐of‐package warning labels and restrictions on the marketing of unhealthy foods including via packing. Longitudinal data suggest this measure has been associated with reduced purchasing of these foods and nutrients of concern.^92^ Previous studies have shown that children are frequently exposed to alcohol marketing via product packaging^93^ and, similar to food, alcohol packaging captures attention, creates product appeal, and contributes to shaping of perceptions about the product and drinking experiences in young adults.^94^ More research is needed to improve understanding of the role of the brand in contributing to the behavioral outcomes associated with exposure to on‐pack marketing for food and alcohol.

Four studies in this review explored brand marketing using sports sponsorship stimuli, with mixed results. Sponsorship of major global sporting events by companies selling alcohol and unhealthy foods is commonplace,^95^ as is the use of athlete endorsement,^44^ which is perhaps unsurprising given the opportunity these present for brands to gain immense exposure and to benefit from affect transfer as the positive emotional associations evoked by sport and sporting celebrities are transmitted to brands and products.^96^ Further, sports celebrity endorsement within food marketing has been shown to influence food choice and intake in children.^97^ It has also been observed that alcohol brands use sport‐linked social media strategies to generate engagement and amplify and augment the connections between the products and the spectator experience.^98^ Restricting sports sponsorship by unhealthy food and alcohol brands has been proposed as an effective public health measure to promote dietary health^99^ and reduce alcohol harms.^100^ However, the current review identified a lack of data on behavioral outcomes of sports‐based food and alcohol brand marketing, such as purchasing or consumption. A similar gap has been noted previously for product‐based stimuli,^101^ so further research is required to meet these gaps in evidence and understanding.

The lack of research on the impact of brand‐only marketing for food and alcohol brands on digital platforms was notable. Spend on digital advertising is forecast to show continued growth in the coming years,^102^ and unhealthy food and alcohol brands are demonstrating ever‐increasing presence across social media and other youth‐dominated online platforms.^103^, ^104^ Businesses are already using mainstream synchronous digital experiences (such as videogame livestreaming platforms^105^, ^106^) as brand extensions, and this is predicted to grow as immersive reality technology develops.^107^ Therefore, there is a clear need for research to better reflect the contemporary digital marketing ecosystem, which could be guided by new conceptual frameworks developed to integrate strategies common to digital platforms into established theoretical models relevant to consumptive behaviors.^108^

Marketers recognize that a strong brand is key to long‐term business success, and brand‐building activities ‐ including brand‐only marketing ‐ are an integral part of achieving that goal.^109^ Unhealthy food and alcohol are highly branded commodities^39^, ^110^ and those brands have meaningful salience to consumers which is critical for brand loyalty and equity.^52^, ^111^ It stands to reason, therefore, that brand marketing of food and alcohol should also be a direction for public health policy actions seeking to build on restrictions on product‐specific marketing to improve population health.^112^ Currently, no global or national government policy explicitly addresses brand marketing for unhealthy products linked to diet‐related non‐communicable diseases. Existing policies regulate exclusively at the product level or via dated broader bans on commercial communications to children^15^ or rely on ineffective industry codes.^19^ A policy to be implemented in the UK in October 2025 is based on legislation that makes no reference to brand advertising, but the frontline regulator's guidance indicates that advertisements will only be restricted if they can be identified as being for a specific unhealthy product.^113^

Regulating brand‐only marketing activity is not without its challenges, as regulating at a brand level may remove incentives for companies to reformulate their products to have healthier nutritional profiles^114^ and there is currently no accepted method for classifying if a brand is healthy or not. Potential approaches include applying a nutrient profile model to a brand's entire product line,^115^ restricting brands based on the proportion of their sales that come from unhealthy products; restricting brands associated with categories of products considered unhealthy (e.g., fast food) and/or consumed in excess by children; requiring a healthier product to be prominently shown on all marketing communications; and restricting specific features of marketing communications that are synonymous or closely associated with an unhealthy product. Importantly, marketing from brands associated with unhealthy food categories increases children's desire to consume unhealthy foods, even when the advertised product is healthy.^116^, ^117^ However, implemented policies whereby brands can only advertise if they include policy‐compliant products have been found to reduce the purchasing of unhealthy foods.^118^ There is further complexity in relation to the marketing of no or low alcohol (‘NoLo’) products, including a need to understand if exposure contributes to addition or substitution (NoLo products being used on top of or in place of full‐strength alcohol ones).^119^ Similarly, regulatory models for food marketing based on nutrient profiling will often allow advertising of zero‐sugar beverages, seeking to drive reformulation and reduced sugar sales,^120^ although there is some evidence to suggest that advertising spending on these beverages increases demand for both this version and the regular (greater sugar) counterpart.^121^

### 4.1 Strengths and limitations

This review has some strengths and limitations. Strengths include the pre‐registration, and the robust methodology used (e.g., comprehensive literature review, independent bias assessments using Cochrane tools). In terms of limitations, the review synthesizes a body of literature that is highly heterogeneous and of mixed quality and has highlighted several gaps where research is needed to inform policy progress in this space. First, there is a need for better quality studies, particularly RCTs to demonstrate causality and provide evidence of greater certainty to underpin guidelines and policies.^122^ Second, more studies should seek to measure actual consumption behaviors, and the longer‐term effects and distal harms associated with food and alcohol brand marketing exposure. This appears pertinent, given concerns about the cumulative effects of marketing^123^ and that evidence supports a hierarchy of effects pathway from marketing exposure to change in body weight (and associated risk of noncommunicable disease).^124^, ^125^ These additional studies may also facilitate subgroup analyses to explore and potentially explain the high heterogeneity identified in the current quantitative synthesis. This review did not consider all outcomes of the hierarchy of effect model, and therefore future research should focus on the cognitive (e.g., awareness, recognition) and affective (e.g., liking, emotion) outcomes to provide a comprehensive understanding of the impacts of brand‐only marketing.

The relative lack of research on the influence of advertising on adults is a notable gap,^57^, ^58^ especially considering that the power of marketing extends beyond immediate behavioral effects. Brand‐building, in particular, is aimed at creating future demand by driving brand awareness and purchase consideration, which later marketing efforts can convert into sales.^109^ To fully understand the mechanisms underlying this process, research needs to map how brand marketing shapes attitudes and cognition which will likely predict future consumption.^21^ It is also important to acknowledge with this research is that even small effects can have substantial consequences at the population level, especially when considered at scale and over time.^126^, ^127^ Although qualitative evidence suggests that food marketing influences brand awareness and attitudes,^128^ and a recent large‐scale study (209 participants) found that adolescents recognized the impact of advertisements on their brand and product awareness, there is a significant shortage of quantitative data, particularly regarding brand‐only marketing. Addressing this gap would enhance our mechanistic understanding of marketing's effects, including the role of emotion, which has been shown to explain more than twice the variance in changes in brand interest and purchase intent compared to explicit brand attitudes.^129^

Third, gender differences in advertising exposure and the impact of gender‐specific brand marketing strategies appear underexplored in the literature to date. In the current review, one study found girls consumed more at the branded versus the unbranded meal whereas boys ate a similar amount across both conditions^71^ but the reasons for this are unclear. Previous studies have shown that adolescent boys and girls see similar volumes of food marketing in social media, but there are significant differences in the products and marketing techniques they are exposed to.^130^ Exploring the role of gender in food brand marketing approaches could be useful to inform intervention strategies. Further, female‐targeted alcohol marketing, including the feminisation of alcohol brands and products (often perpetuating stereotypes using imagery such as the color pink and lifestyle messages that focus on women's friendships and themes of motherhood and beauty) has been implicated in recent increases in alcohol consumption in women^131^ and is said to be contributing to a widening of health and social inequalities.^132^ This is particularly problematic given the ‘risk severity paradox’ whereby despite often drinking less, females can be more vulnerable to experiencing alcohol‐related harms.^133^ Consistent with this, the evidence does suggest that marketing has the biggest effect on the most vulnerable, whether that be children with overweight showing a greater magnitude of response to food marketing exposure^85^ or the enhanced impact of alcohol marketing on young people with existing alcohol issues or lower digital literacy.^134^ Public health policies should seek to reduce these inequalities.

## 5 CONCLUSION

Overall, the findings of this systematic review and meta‐analysis suggest that brand marketing for food and alcohol can influence preference, choice, and purchase intent, but conclusions must be tentative given the limited available evidence and its mixed quality. Several research priorities have been identified, where additional evidence and greater understanding are required to inform public health policy progress toward effective restrictions of the commercial determinants of health. Greater protections, particularly for the most vulnerable, are needed to tackle excess consumption of unhealthy food and alcohol and the associated adverse health outcomes globally.

## AUTHOR CONTRIBUTIONS

EB was responsible for the systematic review, wrote the manuscript, was involved in the interpretation of results. MW, MM, FC, AJ, and AK were involved with the systematic review and the interpretation of results. ND was responsible for the statistical analyses, wrote the manuscript, and was involved in the interpretation of results. ND, MW, RE, AF, LM, and AJ accessed and verified the data. All authors were involved in devising and agreeing the final protocol for this work, had full access to all the data in the study, had final responsibility for the decision to submit for publication, reviewed and commented on the draft manuscript, and approved the submission of the final manuscript.

## CONFLICT OF INTEREST STATEMENT

The authors declare no conflicts of interest.

## Supporting information


**Table S1** Included studies assessing food brand marketing with children (n = 7)
**Table S2.** Included studies assessing food brand marketing with adults (n = 9)
**Table S3.** Included studies assessing alcohol brand marketing with adults (n = 3)
**Table S4.** Study details and risk of bias/quality assessments
**Figure S1.** Risk of bias assessments RCTs
**Table S5.** Risk of bias assessments NRS
**Figure S2.** Forest plot multi‐level meta‐analysis for studies examining consumption as a continuous outcome (n = 5)
**Table S7.** Example search
**Table S8.** Full exclusion criteria (in order of importance)
**Table S9.** Outcome definitions
**Table S10.** Author contact for missing outcome data
