# Supplementary material for: Impact of food, beverage, and alcohol brand marketing on consumptive behaviors and health in children and adults: A systematic review and meta‐analysis
Source: Obes Rev. 2025 Apr 14;26(9):e13932. doi: 10.1111/obr.13932 (PMC12318912; doi:10.1111/obr.13932)
Supplement: Supplementary file 1 — Table S1 Included studies assessing food brand marketing with children (n = 7) Table S2. Included studies assessing food brand marketing with adults (n = 9) Table S3. Included studies assessing alcohol brand marketing with adults (n = 3) Table S4. Study details and risk of bias/quality assessments Figure S1. Risk of bias assessments RCTs Table S5. Risk of bias assessments NRS Figure S2. Forest plot multi‐level meta‐analysis for studies examining consumption as a continuous outcome (n = 5) Table S7. Example search Table S8. Full exclusion criteria (in order of importance) Table S9. Outcome definitions Table S10. Author contact for missing outcome data [file OBR-26-e13932-s001.pdf]

Supplementary Information

Impact of food, beverage, and alcohol brand marketing on consumptive behaviours and health in children and adults: A systematic review and meta-analysis

Emma Boyland[1], Nicholas Davies[1], Moon Wilton[1], Andrew Jones[2], Michelle Maden[3], Ffion Curtis[3], Rebecca Evans[1], Amy Finlay[1], Lauren McGale[4], Caroline Cerny[5], Nika Pajda[5], Abigail K Rose[2]

[1] Department of Psychology, University of Liverpool, Liverpool, UK

[2] Liverpool John Moore's University, Liverpool, UK

[3] Liverpool Reviews and Implementation Group, University of Liverpool, Liverpool, UK

[4] Edge Hill University, Ormskirk, UK

[5] Bite Back 2030, London, UK

Correspondence to:

Prof. Emma Boyland, Department of Psychology, University of Liverpool, Liverpool, L69 7ZA, UK.

Email: eboyland@liverpool.ac.uk.

<u>Contents</u>

Tables S1-S3	Characteristics of included studies
Table S4	Study details and risk of bias/quality assessments
Figure S1	Risk of bias assessments RCTs
Table S5	Risk of bias assessments NRS
Figure S2	Forest plot multi-level meta-analysis for studies examining consumption as a continuous outcome (n=5)
Table S6	Publication bias tests (trim and fill analyses)
Table S7	Example search
Table S8	Full exclusion criteria (in order of importance)
Table S9	PICO table

Table S1 Included studies assessing food brand marketing with children (n=7)

Reference, country of origin, study design	Design, setting	Population characteristics	Commodity category and information	Marketing type and condition(s)	Outcome type(s), outcome definition	Results
Dixon (2018a)[74], Australia	Randomised, between subjects, laboratory	N=1124 children, 48.7% female, age range 5-10, mean age 7.2 (±0.9)<br><br>High SES (43.8%), medium SES (48.3%), low SES (7.9%)	Food<br><br>Unhealthy food (Nutri-Grain, Milo, McDonalds, KFC, Gatorade, Powerade) brands, healthier food (Weet-Bix, Cheerios, Sushi-Sushi, Subway, Mount Franklin, Cool Ridge) brands.<br><br>Unhealthy food brands were determined by authors as being for foods that contained more kilojoules and more fat, sugar, and/or sodium per 100g/100ml than the healthier comparison brand.	Sponsorship (digital)<br><br>(A) non-food branding (control)<br>(B) Unhealthy food branding sponsor<br>(C) Healthier food branding<br>(D) Obesity prevention campaign branding	Preference<br><br>Brand preference (% choosing unhealthy/healthier sponsors)	No significant difference in % choosing unhealthy sponsors between unhealthy (33%) and non-food sponsorship conditions (40.1%) (OR=0.74 [95%CI=0.51, 1.06], p=.096).<br><br>No significant difference in % choosing healthier sponsors between healthier (25.4%) and non-food sponsorship conditions (21.8%) (OR=1.22 [95%CI=0.82, 1.81], p=0.32).<br><br>Reduced preference for

Reference, country of origin, study design	Design, setting	Population characteristics	Commodity category and information	Marketing type and condition(s)	Outcome type(s), outcome definition	Results
						unhealthy sponsor brands between healthier (31.4%) and non-food sponsorship conditions (40.1%) (OR=0.67 [95%CI=0.46, 0.96), p=.027).
Elliot (2013)[71], Canada	Randomised, mixed (within/between subjects), laboratory	N=65 children, 55.4% female, age range 3-5, mean age 3.8 (±0.7)<br><br>White ethnicity (75.4%)	Food<br>Various items<br><br>Brands: McDonalds and Starbucks	Packaging<br><br>All participants tasted food pairs presented in McDonalds branded packaging and either:<br><br>(1) Plain unbranded packaging<br>(2) Colourful unbranded packaging<br>(3) Starbucks branded packaging	Preference<br><br>% participants who preferred McDonalds (vs no preference or preferred alternative)	Children preferred food in McDonalds branded packaging compared to plain packaging (p<.009).<br><br>There were no significant differences in preference between the colourful or Starbucks branded conditions compared to McDonald's packaging (p>.404)

Reference, country of origin, study design	Design, setting	Population characteristics	Commodity category and information	Marketing type and condition(s)	Outcome type(s), outcome definition	Results
Keller (2012)[69] Study 1; Forman (2009)[70], USA	Randomised, within subjects, laboratory	N=43 children, 60.5% female, age range 4-6, mean age 5.9 (±0.9)<br><br>N=20 overweight children<br><br>45% low income families	Food<br><br>Foods that are commonly advertised to children including Kraft (Kraft Foods), Del Monte (Del Monte), Oreo (Kraft Foods), Yoplait (General Mills), Lays (Frito Lay), Kool Aid (Kraft Foods), Nesquik (Nestlé).	Packaging<br><br>(1) Products served in original branded packaging<br><br>(2) Products served in plain unbranded packaging	Consumption<br><br>Food intake (kcal)	No differences in food intake at the branded versus unbranded test meals (p=.80).<br><br>Significant subgroup effects by weight status.
Keller (2012)[69] Study 2, USA	Randomised, within subjects, laboratory	N=41 children, 49% female, mean age 8.4 (±0.5)<br><br>N=19 overweight children<br><br>65% African American or Hispanic American	Food<br><br>Popular fast food brand (not named)	Packaging<br><br>(1) Products served in original branded packaging<br><br>(2) Products served in plain unbranded packaging	Consumption<br><br>Food intake (kcal)	Children ate more at the branded (M=739.0, SD=302.9) compared to the unbranded meal (M=730, SD=326.4; p.07).<br><br>Child weight status did not interact with brand condition (p=.80).

Reference, country of origin, study design	Design, setting	Population characteristics	Commodity category and information	Marketing type and condition(s)	Outcome type(s), outcome definition	Results
						Boys ate similar amounts at the branded versus unbranded meals (p=.66) whereas girls ate around ~100 kcal more (p<.05).
McGale (2016)[72] Study 1, UK	Randomised, mixed (within/between subjects), laboratory	N=40 children, 57% female, age range 4.1-8.7, mean age 7.2 (±1.1)<br><br>BMI 15.9 (±0.2)<br><br>88% White ethnicity, 57% graduate (parent education level)	Food<br><br>Snack brands (n=3) Cheestrings (Kerry Foods), Pom-Bear potato snacks (Intersnack), Coco Pops snack bar (Kellogg's)	Packaging<br><br>(1) Food items served in packaging with brand equity characters<br><br>(2) Food items served in plain unbranded packaging	Choice<br><br>Final snack choice (% choosing brand equity products)<br><br>Preference<br><br>Ratings of taste (scale from -5 [prefers unbranded] to +5 [prefers branded]).	Children were more likely to choose brand equity character food items (73%) than unbranded items $\chi^2(1)=13.07$, p<.001<br><br>Average liking scores were 0.14 (±0.42) indicated a preference for the food items with brand equity characters (Z=-2.537, p=.01).
McGale (2016)[72] Study 2, UK	Randomised, mixed (within/between	N=129 children, 46% female, age range 4.0-8.9,	Food<br><br>Snack brands (n=3)	Packaging	Choice	Children were more likely to choose food items with an

Reference, country of origin, study design	Design, setting	Population characteristics	Commodity category and information	Marketing type and condition(s)	Outcome type(s), outcome definition	Results
	subjects), laboratory	mean age 6.9 (±1.1)<br><br>BMI 16.4 (±2.0)<br><br>94% White ethnicity, 43% graduate (parent education level)	Cheestrings (Kerry Foods), Pom-Bear potato snacks (Intersnack), Coco Pops snack bar (Kellogg's)	(1) Food items served in packaging with incongruent characters (e.g. Pom Bears crisps with Coco the monkey)<br><br>(2) Food items served in plain unbranded packaging	Final snack choice (% choosing brand equity products)<br><br>Preference<br><br>Ratings of taste (scale from -5 [prefers unbranded] to +5 [prefers branded]).	incongruent brand character (58%) than unbranded items $\chi^2(1)=11.56$, $p=.001$.<br><br>However, when asked to make a final snack selection no difference was found ($\chi^2(1)=0.01$, $p=.935$).<br><br>Children preferred food items with brand equity characters compared to plain packaging (Z-3.82, $p<.001$).
Robinson (2007)[73], USA	Randomised, within subjects, laboratory	N=63 children, 55.6% female, age range 3.5-5.4, mean age 4.6 (±0.5)	Food<br><br>McDonalds branded food items (n=5)	Packaging<br><br>Identical food/beverage items either:	Preference<br><br>Taste preference scores (-1 unbranded, +1 branded)	Preference scores were greater than zero (M=0.37, SD=0.45) indicating children preferred the taste of branded products ($p<.001$).

Reference, country of origin, study design	Design, setting	Population characteristics	Commodity category and information	Marketing type and condition(s)	Outcome type(s), outcome definition	Results
		55.6% Latino/Hispanic ethnicity		(1) McDonalds branded packaging<br><br>(2) Plain unbranded packaging		

[1-2]Indicates citations with multiple studies, RR=Relative risk, OR=Odds ratio, M=mean, SD=standard deviation

Table S2 Included studies assessing food brand marketing with adults (n=9)

Reference, country of origin, study design	Design, setting	Population characteristics	Commodity category and information	Marketing type and condition(s)	Outcome type(s), outcome definition	Results
Conti-Silva (2019)[75], Brazil	Non-randomised, within subjects, laboratory	N=85 adults, 71% female, age range 18-30	Food<br><br>N=4 commercial probiotic fermented milk brands (only listed as A-D)	Packaging<br><br>(1) Blind test<br>(2) Brand information provided<br>(3) Brand information and probiotic claim provided	Preference<br><br>Overall liking (measure of overall sensory acceptance)	Brand exposure increased degree of liking for product B (p=.018), product A (RR=1.29 [95%CI=0.28, 0.99], p<.05) and product D (RR=1.48 [95%CI=0.34, 0.81], p<.05).<br><br>No significant difference for product C (RR=1.00 [95%CI=0.19, 1.44], p>.05).
Dixon (2018b)[78], Australia	Randomised, between subjects, laboratory	N=1132 adults, 53% female, age range 18-24, mean age 21.0 (±2.1)<br><br>Healthy weight (69.8%), overweight (20.0%), obese (10.1%)	Food<br><br>Brands not named, in breakfast cereal, takeaway food, and non-alcoholic beverage categories.<br><br>Healthiness	Sponsorship (digital)<br><br>(1) Unhealthy food/beverage brand sponsor<br>(2) Healthier food/beverage brand sponsor<br>(3) Non-food brand sponsor	Preference<br><br>Brand preference (% choosing unhealthy/healthier sponsors)	No significant difference in % choosing unhealthy sponsors between unhealthy (31.3%) and non-food sponsorship conditions (33.7%) (OR=0.89 [95%CI=0.63, 1.27], p=.531).<br><br>Increased preference for healthier food sponsor brands between healthier

Reference, country of origin, study design	Design, setting	Population characteristics	Commodity category and information	Marketing type and condition(s)	Outcome type(s), outcome definition	Results
		High SES (38.6%), medium SES (36.7%), low (24.6%)	based on author judgement of the overall nutritional profile of the majority of foods within that brand.	(4) Obesity prevention campaign sponsor		(43.9%) and non-food sponsorship conditions (34.7%) (OR=1.47 [95%CI=1.05, 2.07], p=.026).
Farrar (2022)[79] Study 1, UK	Randomised, between subjects, field	N=170 adults, 32% female (control group) 45%-47% female (intervention groups), mean age 25.1 (±9.8) (control group), 24.0 (±7.3)-27.8 (±11.2) (intervention groups) BMI: 23.9 (±3.4) (control), 32.1 (±3.5) and 25.2 (±4.7) (intervention groups)<br><br>Majority educated up to A level (46-60%)	Food<br><br>Marks & Spencer (M&S) fruit and nut selection<br><br>Mars bar	Digital<br><br>(1) M&S logo prime<br>(2) Mars logo prime<br>(3) no food related logo shown	Choice<br><br>Snack choice (M&S, Mars, none)	Participants exposed to the M&S prime or the Mars prime were no more likely than control group to select the M&S or Mars-related food product (OR=0.93 [95%CI=0.44, 1.98], p>.05; OR=0.97 [95%CI=0.45, 2.07], p>.05).
Farrar (2022)[79] Study 2, UK	Randomised, between	N=158 adults, mean age 21.8(± 6.3)	Food	Digital	Choice	Mean unhealthy food choice scores were similar in the control

Reference, country of origin, study design	Design, setting	Population characteristics	Commodity category and information	Marketing type and condition(s)	Outcome type(s), outcome definition	Results
	subjects, laboratory	(control) 20.8(± 6.8) (intervention)<br><br>BMI: 23.3(±5.4) (control), 24.5(±6.4) (prime)<br><br>Majority educated up to A levels (70-78%)	Author defined as unhealthy brands: McDonald's, Ben & Jerry's, Magnum, Cadbury's, Thornton's, Mr Kipling	(1) Unhealthy food related logos prime<br>(2) Non-food related logos (clothing brands)	Unhealthy food choice scores	(M=2.88, SD=1.41) and priming conditions (M=2.96, SD=1.4; t(156)=-0.36, p=.72).
Kim (2013)[76], USA	Non-randomised, within subjects, laboratory	N=108 adults, age range 19-65	Food<br><br>Unnamed chocolate milk brands (n=7)	Packaging<br><br>(1) Blind product evaluation<br><br>(2) Branded product evaluation	Purchase<br><br>Purchase intent scores	Purchase intention of national brand milks was higher after brand or package exposure.<br><br>The effect of brand or packaging exposure on regional and store brand milk was variable.
Werle (2016)[67] Study 1, France	Randomised, between subjects, laboratory	N=166 adults, 66.5% female, mean age 20.43<br><br>BMI 21.05	Food<br><br>M&Ms	Packaging<br><br>(1) Branded packaging<br><br>(2) Plain unbranded packaging	Consumption<br><br>Intended consumption (7 point Likert scale)	Participants exposed to the plain packaging had lower intentions to consume the product (M=2.52, SD=1.95) compared to those exposed to the branded product (M=4.46,

Reference, country of origin, study design	Design, setting	Population characteristics	Commodity category and information	Marketing type and condition(s)	Outcome type(s), outcome definition	Results
						SD=1.98; F(1,165)=42.28, p=.001).
Werle (2016)[67] Study 2, France	Randomised, between subjects, laboratory	N=77 adults, 45.5% female, mean age 20 (±0.92)	Food<br><br>M&Ms	Packaging<br><br>(1) Branded packaging<br><br>(2) Plain unbranded packaging	Consumption<br><br>Food intake (grams)	No differences in consumption between branded (M=28.83, SD=12.39) and unbranded (M=25.21, SD=11.37) conditions F(1,76)=1.78, p=.187.<br><br>Males in the plain packaging condition consumed more than males exposed to branded packaging.
Werle (2016) [67], Study 3, France	Randomised, between subjects, laboratory	N=66 adults, 55.6% female, mean age 20.65 (±1.35)	Food<br><br>M&Ms	Packaging<br><br>(1) Original branded packaging<br><br>(2) Lighter/low fat branded packaging<br><br>(3) Plain unbranded packaging	Consumption<br><br>Food intake (grams)	Signirifcant main effect of condition on consumption (F(2,98)=3.45, p=.036). Participants exposed to the plain packaging (M=22.88, SD=12.48) and the low fat packaging (M=24.53, SD=11.21) ate more than those exposed to original packaging (M=18.34, SD=11.37).

Reference, country of origin, study design	Design, setting	Population characteristics	Commodity category and information	Marketing type and condition(s)	Outcome type(s), outcome definition	Results
						Males exposed to plain packaging and low fat packaging ate more than males exposed to original packaging.<br><br>Females exposed to low fat packaging ate more than females exposed to original packaging.
Wlodarska (2019)[77], Poland	Non-randomised, between subjects, laboratory	N=96 adults, 64.60% female	Beverage<br><br>Unnamed apple juice brands (n=8)	Packaging<br><br>(1) Blind evaluation<br><br>(2) Brand and packaging exposure	Preference<br><br>Consumer liking scores (9 point hedonic scale)	Consumer liking scores were lower in the blind condition (range=4.1-6.2) compared to the branded condition (5.8-7.1).

[1-2]Indicates citations with multiple studies, RR=Relative risk, OR=Odds ratio, M=mean, SD=standard deviation

Table S3 Included studies assessing alcohol brand marketing with adults (n=3)

Reference, country of origin, study design	Design, setting	Population characteristics	Product/brand information	Advertising type and condition(s)	Outcome type(s), outcome definition	Results
Dos Santos (2020)[81], Chile	Randomised, between subjects, laboratory	N=90 adults	Alcohol<br><br>Beer brands (n=3) Heineken, Becker, Cristal	Sponsorship (digital)<br><br>(1) Alcohol sponsor<br>(2) Non-alcohol sponsor (congruent)<br>(3) Non-alcohol sponsor (incongruent)	Consumption<br><br>Intention to consume alcohol	No significant difference between alcohol and non-alcohol sponsorship conditions on intention to consume alcohol.
Dos Santos (2021)[80], Chile	Randomised, between subjects, laboratory	N=180 adults	Alcohol<br><br>Beer brands (n=3) Heineken, Becker, Cristal	Sponsorship (digital)<br><br>(1) Alcohol sponsor<br>(2) Non-alcohol sponsor (congruent)<br>(3) Non-alcohol sponsor (incongruent)	Purchase<br><br>Intention to purchase sponsor products	Intention to purchase was higher in the alcohol sponsorship condition (M=2.88, SD=1.06) compared to the congruent (M=2.63, SD=1.00) and incongruent (M=2.59, SD=1.08) conditions.
Kelly (2018)[82], Australia	Randomised, between subjects, online	N=244 adults, 56.6% female, mean age 23.07	Alcohol<br><br>Beer brands (n=2)	Advergame<br><br>(1) Advergame features alcohol branding	Choice<br><br>Brand choice	Health messages in conjunction with alcohol (vs non-alcohol) sponsored advergames did not affect brand choice $\chi^2(286)=323.23$, p=.064

	XXXX Gold, Budweiser	(2) Advergame features non-alcohol related branding	

Table S4 Study details and risk of bias/quality assessments

**Citation details**			**Study details**				**Outcomes reported**				**Quality/risk of bias assessment**	
**Author**	**Year**	**Country**	**Design**	**Product**	**Sample**	**Marketing format**	**Consumption**	**Choice**	**Purchase**	**Preference**	**ROB**	**NOS**
Conti-Silva	2019	Brazil	NRS	Beverage	Adult	Packaging				X		3
Dixon	2018a	Australia	RCT	Food & beverage	Child	Sponsorship				X	Some concerns	
Dixon	2018b	Australia	RCT	Food & beverage	Adult	Sponsorship				X	Some concerns	
Dos Santos	2020	Chile	RCT	Alcohol	Adult	Sponsorship	X				High	
Dos Santos	2021	Chile	RCT	Alcohol	Adult	Sponsorship			X		High	
Elliot	2013	Canada	RCT	Food	Child	Packaging				X	Some concerns	
Farrar	2022	UK	RCT	Food	Adult	Digital		X			Some concerns (both studies)	
Keller; Forman	2012; 2009	USA	RCT	Food & beverage	Child	Packaging	X				Some concerns	

Citation details			Study details				Outcomes reported				Quality/risk of bias assessment	
**Author**	**Year**	**Country**	**Design**	**Product**	**Sample**	**Marketing format**	**Consumption**	**Choice**	**Purchase**	**Preference**	**ROB**	**NOS**
Keller	2012	USA	RCT	Food	Child	Packaging	X				Some concerns (both studies)	
Kelly	2018	Australia	RCT	Alcohol	Adult	Advergame		X			Some concerns	
Kim	2013	USA	NRS	Beverage	Adult	Packaging			X			3
McGale	2016	UK	RCT	Food	Child	Packaging		X		X	Some concerns (both studies)	
Robinson	2007	USA	RCT	Food & beverage	Child	Packaging				X	Some concerns	
Werle	2016	France	RCT	Food	Adult	Packaging	X				Some concerns (3 studies)	
Wlodarska	2019	Poland	NRS	Beverage	Adult	Packaging				X		2

Figure S1 Risk of bias assessments RCTs

Lead Author & Year	D1	D2	D3	D4	D5	Overall
Dixon 2018	+	+	+	!	!	!
Dixon 2018	+	+	+	!	+	!
Dos Santos 2020	+	+	-	!	!	-
Dos Santos 2021	+	+	-	!	!	-
Elliot 2013	+	+	+	!	!	!
Farrar 2022 (Study 1)	+	+	+	!	+	!
Farrar 2022 (Study 2)	+	+	+	!	+	!
Keller 2012 (Study 1)	+	+	+	!	!	!
Keller 2012 (Study 2)	+	+	!	!	!	!
Kelly 2018	+	+	!	!	!	!
McGale 2016 (Study 1)	+	+	!	!	!	!
McGale 2016 (Study 2)	+	+	!	!	!	!
Robinson 2007	+	+	+	!	+	!
Werle 2016 (Study 1)	+	+	!	!	!	!
Werle 2016 (Study 2)	+	+	!	!	!	!
Werle 2016 (Study 3)	+	+	!	!	!	!

+ Low risk

! Some concerns

- High risk

D1 Randomisation process

D2 Deviations from the intended interventions

D3 Missing outcome data

D4 Measurement of the outcome

D5 Selection of the reported result

Table S5 Risk of bias assessments NRS

	Selection			Comparability	Outcome		
**Study name**	**Representativeness of the sample**	**Non-respondents**	**Ascertainment of the exposure (risk factor)**	**Comparability**	**Assessment of the outcome**	**Statistical test**	**Total score**
Conti-Silva 2019	c	c	c	a	c	a	3
Kim 2013	b	c	c	a	c	b	3
Wlodarska 2019	c	c	c	a	c	b	2

a, b, c, d = these letters denote the study quality rating for each item across all domains on the NOS scale with ‘a’ indicating the highest quality

Figure S2. Forest plot multi-level meta-analysis for studies examining consumption as a continuous outcome (n=5)

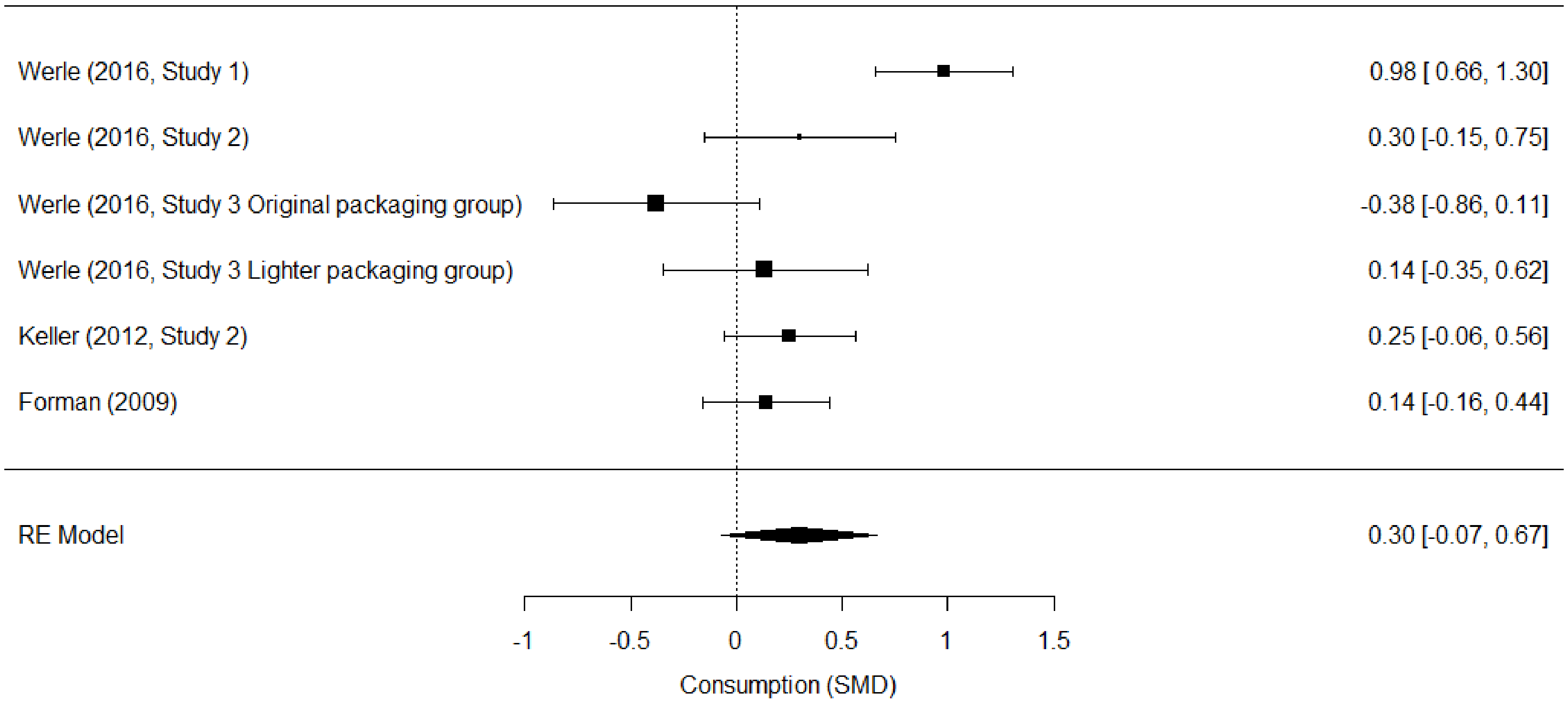

Table S6. Publication bias tests (trim and fill analyses)

Pre-registered analyses	Funnel plot asymmetry (z)	Funnel plot asymmetry (p)	N "missing" effects	Pooled effect estimate, SMD (95%CI)	p
Consumption	-1.22	.2227	2	0.42 (0.07, 0.77)	.0180

**p*<.05 ***p*<.01 ****p*<.001

Table S7 Example search

Ovid MEDLINE(R) ALL <1946 to February 19, 2024>

1 exp Food/ or exp Food Industry/ or beverages/ or carbonated beverages/ or energy drinks/ or "fruit and vegetable juices"/ or exp milk/ or exp milk substitutes/ or exp Tea/ or exp Teas, Herbal/ or exp alcoholic beverage/ 1632899

2 (food* or diet* or snack* or nutrition* or fast-food* or beverage* or drink* or tea or milk or juice or alcohol* or nonalcohol* or non-alcohol* or low-alcohol*).ti. 789212

3 Diet/ 190742

4 1 or 2 or 3 2159455

5 Direct-to-Consumer Advertising/ or Advertising as Topic/ 16147

6 exp Marketing/ 37954

7 (advert* or advergam* or sponsor* or promot* or market* or commercial or commercials).ti. 325978

8 5 or 6 or 7 348271

9 4 and 8 30033

10 ((food* or diet* or snack* or nutrition* or fast-food* or beverage* or drink* or tea or milk or juice or alcohol* or nonalcohol* or non-alcohol* or low-alcohol*) adj3 (advert* or advergam* or sponsor* or promot* or market* or commercial or commercials)).ab. 26079

11 9 or 10 51918

12 (brand* or unbrand* or logo* or slogan* or food-brand* or beverage-brand* or alcohol-brand* or alcoholic-brand*).ti,ab. 31310

13 11 and 12 1241

14 limit 13 to (english language and yr="2004 -Current") 1112

15 exp animals/ 26999032

16 human/ 21799747

17 15 not 16 5199285

18 14 not 17 1024

19 (letter or editorial or comment).pt. 2228507

20 18 not 19 1012

Table S8. Full exclusion criteria (in order of importance)

Reason for exclusion in Covidence	Description of reason
Language	Full text is not provided in English.
Duplicate	Is a duplication of another entry.
Publication type	Not-peer reviewed e.g. book, thesis, commentary, editorial, newspaper article, conference abstract/proceedings.
Publication type	Articles without primary data, e.g. protocol or methodology papers.
Study design	Reviews of any kind without primary data (scoping, narrative or systematic review – SR used only to cross-check primary articles).
Study design	Non-comparative study.
Population	Studies exclusively involving non-human populations.
Population	Studies exclusively involving clinical populations.
Outcomes	Studies that do not report on consumption (or intended consumption), preference (self-report e.g., via checklist), choice (or intended choice), purchase (or intended purchase) or body weight (or BMI/obesity).
Intervention	Studies assessing the impact of marketing for products other than foods, beverages or alcoholic beverages
Intervention	Studies assessing the impact of marketing for foods, beverages or alcoholic beverages that do not expose participants to brand-only, product-free marketing or only include self-reported marketing exposure.
Intervention	Studies assessing the impact of marketing strategies that are outside of the definition provided, e.g. product design, price positioning, distribution channels, business-to-business marketing, lobbying etc.
Intervention	Studies solely assessing effects of non-marketing interventions (e.g. television viewing in general, self-efficacy programmes), or non-commercial campaigns (such as public health education initiatives).
Comparator	Studies without an appropriate comparator. Appropriate comparators include control advertisements or a non-marketing control.<br><br>For a **food/beverage brand** intervention, appropriate comparators would be:<br>(i) food/beverage product with no brand imagery<br>(ii) brand imagery for a generic/supermarket brand of the same product type (e.g., Coca Cola vs Aldi Coke)

(iii) brand imagery for a brand unrelated to consumption (e.g., toys)
(iv) no advertisement (e.g., non-marketing stimulus image).

For an **alcohol brand** intervention, appropriate comparators would be:
(i) alcohol product with no brand imagery
(ii) brand imagery for a generic/supermarket brand of the same product type (e.g., Carlsberg vs Aldi Lager)
(iii) brand imagery for a brand unrelated to consumption (e.g., stationery)
(iv) no advertisement (e.g., non-marketing stimulus image).

Table S9 Outcome definitions

Outcome category	Outcome	Definition of outcome
Diet-related cognitive	Food, beverage and alcoholic beverage preferences	The preferential selection, as measured by quantity or frequency of items selected, or cognitive bias toward product, affected by numerous motives including liking, taste, perceived health benefits and price.
	Food, beverage and alcoholic beverage choice or intended choice	Brand choice, product choice, food/beverage category choice or meal choice, derived from observations of frequency or percent actual choice, or choice behaviour (pointing, computer-based tasks, paper and pen selection).
Diet-related behavioural	Product requests by adults or children (e.g. "pester power") or intended requests	Adults and children requesting, or expressing an intention to request the purchase a particular food, beverage or alcoholic beverage; or children requesting, or expressing an intention to request that parents/caregivers purchase on their behalf, as measured by the volume/frequency of requests
	Food, beverage and alcoholic beverage purchasing/sales (by adults, by children or on behalf of children) or intended purchasing	Acquiring foods, beverages or alcoholic beverages through payment or expressing an intention to acquire such goods in the near future, as measured by volume/frequency of purchase/intended purchase
	Food, beverage and alcoholic beverage consumption or intended consumption	Intake in kcal, kg/ml, nutrients or nutritional quality, inclusive of measurements derived from lab-based studies, food frequency measures and food diaries.
Health-related	Body weight/body mass index/obesity	Adults and children's body weight (kg)/Body Mass Index or obesity (percent or Odds Ratio)

Table S10 Author contact for missing outcome data

Citation	Outcome	Corresponding author	Request (date)	Data provided	Included in meta-analyses
Dos Santos (2020)	Consumption	Manuel Alonso Dos Santos	Email (27.06.24)	No	No
Farrar (2022)	Choice	Stepahanie Farrar	Email (27.06.24)	Yes	No
Keller (2012, Study 1); Forman (2009)	Consumption	Kathleen Keller	Email (27.06.24)	Yes	Yes
Kelly (2018)	Choice	Sarah Jane Kelly	Email (27.06.24)	No	No
Kim (2013)	Preference	M.A. Drake	Email (27.06.24)	No	No
Wldoarska (2019)	Preference	Katarzyna Włodarska	Email (27.06.24)	No	No
McGale (2016)	Preference	Lauren McGale	Email (08.07.24)	Yes	No
